# A Machine Learning Prediction Model for In-Hospital Mortality in Postoperative Patients With Type A Aortic Dissection

**DOI:** 10.31083/RCM49025

**Published:** 2026-08-27

**Authors:** Ming-hao Luo, Xian-tong Cao, Yin-rui Huang, Fei-bai Yao, Guo-wei Tu, Ying Su, Jun-yi Hou, Yi-jie Zhang, Chun-sheng Wang, Hao Lai, Cong Tian, Yana Yuan, Tao Shi, Zhe Luo

**Affiliations:** ^1^Cardiac Intensive Care Center, Zhongshan Hospital, Fudan University, 200437 Shanghai, China; ^2^Shanghai Medical College, Fudan University, 200437 Shanghai, China; ^3^Department of Cardiovascular Surgery, First Affiliated Hospital of Xi’an Jiaotong University, 710063 Xi’an, Shaanxi, China; ^4^Department of Cardiac Surgery, Zhongshan Hospital, Fudan University, 200437 Shanghai, China; ^5^Philips Research China, 201103 Shanghai, China; ^6^Shanghai Key Laboratory of Lung Inflammation and Injury, 200032 Shanghai, China; ^7^Department of Critical Care Medicine, Shanghai Xuhui Central Hospital, Zhongshan-Xuhui Hospital, Fudan University, 200437 Shanghai, China; ^8^Key Laboratory of Multiple Organ Failure (Zhejiang University), Ministry of Education, 310058 Hangzhou, Zhejiang, China

**Keywords:** type A aortic dissection, multiple organ failure, mortality, perioperative medicine, cardiac surgery

## Abstract

**Backgrounds:**

Scoring systems are increasingly used for risk stratification in the postoperative management of type A aortic dissection (TAAD), but no consensus exists regarding the optimal tool. Thus, this study aimed to develop and validate a machine learning-based model to predict in-hospital mortality among postoperative patients with TAAD.

**Methods:**

Data from postoperative TAAD patients treated at two centers were analyzed retrospectively. The primary outcome was in-hospital all-cause mortality. Clustering methods were implemented for feature selection. A random forest algorithm was selected from a range of machine learning methods to develop a postoperative mortality risk prediction tool. Receiver operating characteristic (ROC) curves were employed to assess predictive accuracy and reliability. Model calibration was evaluated by plotting calibration curves. SHapley Additive exPlanations (SHAP) values were calculated to quantify the contribution of each feature. Model performance was compared with the European System for Cardiac Operative Risk Evaluation II (EuroSCORE II).

**Results:**

A total of 983 patients who underwent surgical repair for TAAD were included in the study. Eight features were identified as significant predictors of in-hospital mortality. On postoperative day (POD) 1, the final random forest model achieved an area under the receiver operating characteristic curve (AUROC) of 0.8899 (95% confidence interval (CI): 0.7988–0.9469). External validation on POD 1 yielded an AUROC of 0.8331 (95% CI: 0.7722–0.8871). The model demonstrated significantly higher discriminative performance than EuroSCORE II.

**Conclusions:**

This study developed and primarily validated an accurate machine learning model to predict the all-cause in-hospital mortality of patients with TAAD undergoing surgical repair.

## 1. Background

Risk stratification for postoperative patients with type A aortic dissection (TAAD) is critical. Over the past decade, there has been a growing emphasis on developing and using scoring systems to stratify risk in the management of postoperative patients with TAAD [1,2]. However, consensus regarding the superiority of any specific system remains elusive.

Multiple risk stratification scoring systems have been proposed to date. The European System for Cardiac Operative Risk Evaluation (EuroSCORE) was established in 1995, followed by an updated version, EuroSCORE II [3]. Subsequently, Czerny et al. [4] introduced the German Registry of Acute Aortic Dissection Type A (GERAADA) score. Nonetheless, despite new evidence suggesting promising results, detailed evaluations of these models have generally demonstrated suboptimal predictive accuracy for mortality [5]. Furthermore, external validation of four recently developed scores yielded suboptimal outcomes. Although the GERAADA score yielded the most favorable results, none of the scores achieved adequate discrimination or calibration for predicting early mortality [6]. Additional data suggest that the GERAADA score is less effective than the well-established EuroSCORE II, raising further concerns about the clinical utility of this metric [7].

Machine learning (ML) methods can generate models with superior performance even when based on relatively limited datasets [8,9]. Therefore, this study aimed to develop and validate a robust scoring model that accurately predicts in-hospital mortality among patients undergoing surgical treatment for TAAD.

## 2. Methods

### 2.1 Study Design

The study was conducted in two phases: (1) development and internal testing of an ML model using a cohort from a high-volume center, Zhongshan Hospital, Fudan University (ZS cohort), and (2) external testing of the performance of the model using retrospective data from a second high-volume institution, the First Affiliated Hospital of Xi’an Jiaotong University (XJ cohort).

### 2.2 Patient Population

Retrospective data were collected for postoperative patients with TAAD who underwent surgical repair at Zhongshan Hospital, Fudan University, between January 2020 and December 2022. The external testing cohort from the First Affiliated Hospital of Xi’an Jiaotong University included patients who underwent surgical repair for TAAD between December 2020 and November 2022.

Patients meeting any of the following criteria were excluded: (a) age <18 years; (b) death during surgery or within the first 24 hours postoperatively; (c) receipt of renal replacement therapy before surgery; (d) confirmed neurological conditions on computed tomography or magnetic resonance imaging due to aortic dissection before surgery; (e) records with missing key data.

### 2.3 Data Collection

Perioperative data were extracted from the electronic medical record system and securely stored in the institutional database. Daily laboratory examinations, including complete blood count and liver and kidney function tests, were conducted each morning. Comprehensive records encompassing preoperative demographic characteristics, laboratory test results, surgical data, and the incidence of major postoperative complications were documented. Sequential Organ Failure Assessment (SOFA) and EuroSCORE II scores were calculated by attending physicians according to established methodologies from prior studies [3,10]. For variables measured multiple times per day, mean values were used to indicate the daily status. The primary endpoint was in-hospital all-cause mortality, defined comprehensively as death from any cause.

### 2.4 Data Preprocessing and Feature Selection

We reviewed existing scoring systems and reported risk factors for mortality in TAAD patients, and consulted clinical experts. Subsequently, we selected 156 variables that were consistently measured at both high-volume centers, including demographics, comorbidities, perioperative laboratory results, surgical details, and vital signs. All categorical variables were converted to numerical values using one-hot encoding. Variables with >30% missing data and highly correlated variables (>80%) were also excluded from further feature selection. Missing values for each numerical variable were imputed using multiple imputation.

Given the complexity posed by the low incidence of TAAD and the high dimensionality of the collected predictive variables, we implemented clustering methods for feature reduction, an unsupervised technique widely used for dimensionality reduction in cell type clustering, neuroimaging analysis, and subphenotyping [11,12,13,14]. We fitted the k-means model to reduce dimensionality. To identify the optimal number of clusters (k), the K-means elbow method was used to determine the appropriate number of clusters, the within-cluster sum of squares (SSE) was selected as the primary evaluation metric. 

### 2.5 Model Development

We trained the models using a variety of common ML algorithms on the ZS cohort and compared the respective performances based on key metrics such as area under the receiver operating characteristic curve (AUROC), recall, sensitivity, and others. The random forest model, which achieved the highest AUROC, was selected as the final model. Detailed performance metrics for each model are presented in **Supplementary Table 1**. To minimize the impact of variable scale differences on the performance of algorithms that rely on weighted sums of variables, all variables were normalized using a MinMax scaler.

We employed a 2-fold cross-validation (k = 2) on the ZS cohort to fine-tune the model parameters and prevent overfitting. In K-fold cross-validation, the dataset is randomly split into k folds, with each fold used as a validation set and the remaining folds used to train a model. According to our study, the combination of hyperparameters that yielded the best performance on the validation sets was then evaluated separately on the test set. To address class imbalance, we applied up-sampling using the Synthetic Minority Over-sampling Technique (SMOTE). In classification models, imbalanced datasets occur when classes are not equally represented, leading to biased predictive models that favor the majority class. SMOTE mitigates this problem by generating synthetic samples for the minority class through a three-step process: identifying minority class samples, selecting nearest neighbors, and generating synthetic samples. Through this structured supervised learning approach, we aimed to enhance the predictive accuracy and reliability of the model in clinical settings.

### 2.6 Model Performance and Validation

To rigorously evaluate the performance and robustness of our predictive model, we used ROC curves for both cohorts. In addition to discrimination, model calibration was evaluated using calibration curve plots. SHapley Additive exPlanations (SHAP) values were calculated to quantify the contribution of each feature, with positive SHAP values indicating an increased risk of in-hospital mortality. These analyses can help clinicians select an appropriate threshold for model application based on the trade-off between true and false positives.

### 2.7 Statistical Analysis

Continuous variables are presented as the mean and standard deviation for normally distributed data, or as medians and interquartile ranges (IQR; 25th and 75th percentiles) for skewed data. Categorical variables are expressed as counts and percentages. Comparisons between groups were performed using Student’s *t*-test, Wilcoxon rank-sum test, or Fisher’s exact test, as appropriate. All statistical tests were two-sided, and *p* < 0.05 was considered statistically significant. Statistical analyses were conducted using Python version 3.7.15 (Python Software Foundation, Wilmington, DE, USA).

## 3. Results

### 3.1 Baseline and Perioperative Characteristics

A total of 1005 participants from two centers were initially included in the study (**Supplementary Fig. 1**). A total of 22 patients from the ZS cohort were excluded due to missing data on sex and in-hospital outcomes, resulting in a final sample of 983 participants. Baseline characteristics for the ZS and XJ cohorts are summarized in Table 1 and **Supplementary Table 2**. The two cohorts exhibited similar distributions of age, sex, and preoperative renal function. However, the XJ cohort had significantly higher preoperative D-dimer and total bilirubin levels. Most patients at both centers underwent total aortic arch replacement with elephant trunk implantation, with or without additional procedures. Notably, the ZS cohort had a longer duration of total cardiopulmonary bypass (CPB) and aortic cross-clamping. On the first postoperative day (POD 1), there were notable differences in several clinical parameters between the cohorts, although the SOFA scores were comparable. In terms of outcomes, mortality rates were similar between the two cohorts. Nevertheless, patients in the XJ cohort experienced longer intensive care unit (ICU) and hospital stays, whereas those in the ZS cohort had a longer duration of mechanical ventilation (MV).

**Table 1.** **Demographics and perioperative information**.

		Overall	XJ cohort	ZS cohort	*p*-value
		n = 983	n = 531	n = 452	
Age, median (IQR)		55 (46–64)	55 (48–64)	54 (44–65)	0.260
Male, n (%)		739 (75.3)	403 (76.2*)	336 (74.3)	0.553
BMI, median (IQR)		25.1 (23.0–27.7)	25.0 (23.0–27.7)	25.2 (22.9–28.0)	0.658
Hypertension, n (%)		612 (62.4)	328 (61.9*)	284 (63.0)	0.777
Preoperative values					
	D-dimer, median (IQR)	8.6 (3.0–21.2)	9.3 (3.4–24.0)	6.7 (2.7–18.4)	0.001
	Cr, median (IQR)	76.0 (61.0–104.0)	77.0 (59.0–108.0)	76.0 (64.0–99.2)	0.703
	Hb, median (IQR)	131.0 (118.0–144.0)	130.0 (117.0–143.0)	132.0 (120.0–145.0)	0.325
	TB, median (IQR)	16.6 (11.2–23.2)	18.6 (13.3–26.8)	14.1 (9.4–18.7)	<0.001
Surgical information					
	TAAR, n (%)	905 (92.1)	515 (97.0)	390 (86.3)	<0.001
	CPB duration, median (IQR)	159.0 (140.0–186.0)	149.0 (132.0–172.2)	174.5 (150.0–205.0)	<0.001
	ACD, median (IQR)	87.0 (74.5–105.0)	85.0 (73.0–97.0)	94.0 (77.0–116.0)	<0.001
Clinical values on POD 1					
	EuroSCORE II, mean (SD)	8.2 (3.1)	9.0 (1.3)	7.3 (4.2)	<0.001
	GCS, mean (SD)	14.0 (2.1)	14.3 (1.8)	13.7 (2.3)	<0.001
	SOFA, mean (SD)	7.2 (2.8)	7.1 (2.0)	7.3 (3.5)	0.471
	D-dimer, median (IQR)	14.6 (7.8–22.7)	16.5 (9.2–25.1)	6.8 (4.6–9.7)	<0.001
	BUN, median (IQR)	10.0 (7.7–12.7)	10.1 (8.1–13.5)	9.9 (7.3–12.2)	<0.001
	Cr, median (IQR)	110.0 (80.0–156.0)	94.0 (69.0–138.0)	126.0 (96.0–171.0)	<0.001
	Hb, median (IQR)	105.0 (95.0–114.0)	108.0 (98.0–117.0)	101.0 (90.0–110.0)	<0.001
	TB, median (IQR)	34.0 (22.6–48.2)	39.8 (27.3–53.1)	29.8 (19.5–43.2)	<0.001
	Lactate, median (IQR)	6.1 (3.1–10.0)	8.5 (6.3–11.9)	3.0 (2.0–4.6)	<0.001
Clinical outcomes					
	In-hospital mortality, n (%)	86 (8.7)	45 (8.5)	41 (9.1)	0.829
	MV duration, median (IQR), days	2.0 (1.0–3.3)	1.2 (0.6–3.0)	2.0 (2.0–4.0)	<0.001
	ICU duration, median (IQR), days	5.2 (3.5–8.8)	6.0 (4.0–10.0)	5.0 (3.0–8.0)	<0.001
	Hospital duration, median (IQR), days	15.7 (11.1–20.0)	18.0 (14.0–22.0)	12.0 (9.0–17.0)	<0.001

Cr, serum creatinine; Hb, hemoglobin; TB, total bilirubin; TAAR, total aortic arch replacement with elephant trunk implantation; CPB, cardiopulmonary bypass; ACD, aortic clamping duration; POD, postoperative day; EuroSCORE II, European System for Cardiac Operative Risk Evaluation II; SOFA, Sequential Organ Failure Assessment; GCS, Glasgow Coma Scale; BUN, blood urea nitrogen; MV, mechanical ventilation; ICU, intensive care unit; IQR, interquartile ranges; BMI, body mass index. * The missing values in the Table: two missing values in the XJ cohort of Gender; one missing value in the XJ cohort of hypertension.

### 3.2 Feature Selection

**Fig. 1.**
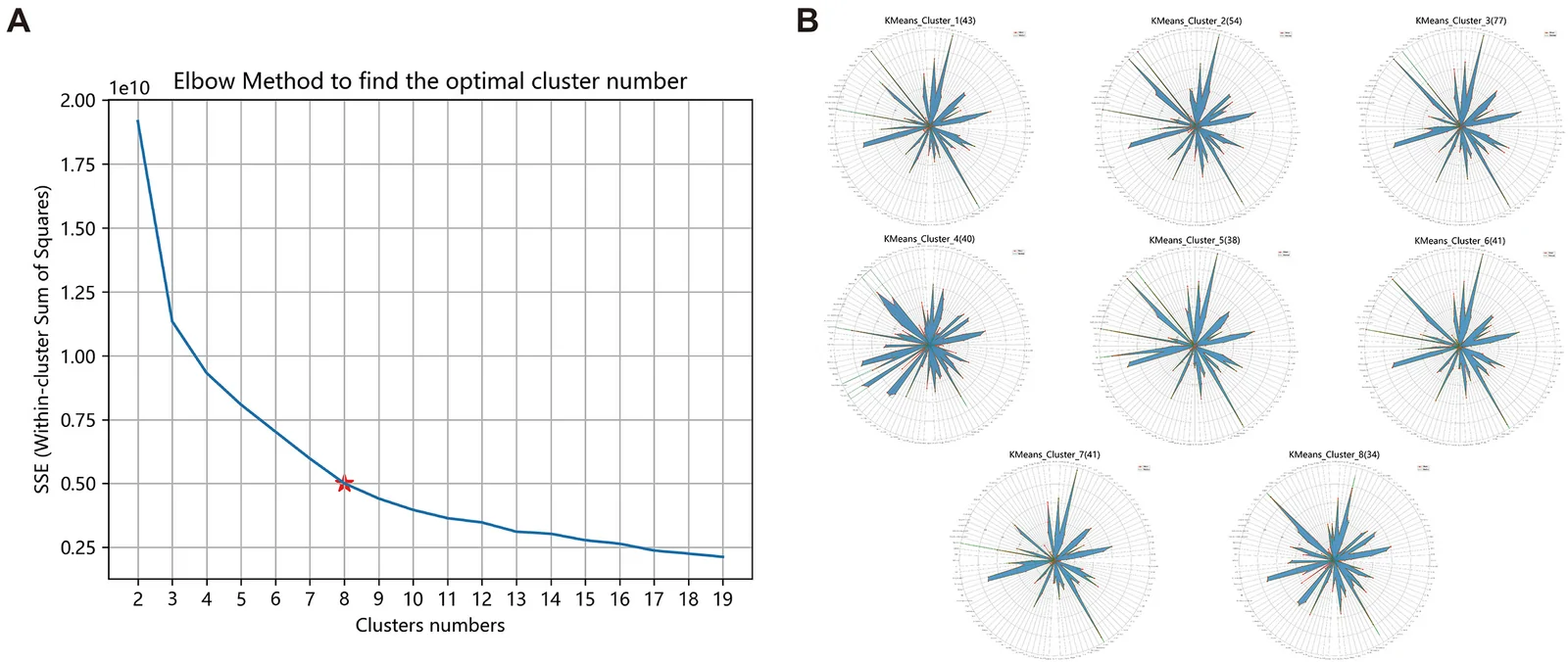
**Feature selection using the cluster methods**. (A) Justification for the chosen number of clusters (k = 8). (B) Radar map for feature selection.

We fitted the k-means model with eight clusters based on the K-means elbow method, ensuring that all deceased and surviving patients were separated into distinct clusters. The summary graph generated by the k-means clustering method illustrated the relative importance and contribution of each feature, as well as the range and hierarchy with respect to model performance. In our analysis, non-survivors in the training set were assigned to 2 of the 8 clusters, as depicted in Fig. 1. Compared with the surviving class, eight features were identified as significant predictors of in-hospital mortality: sex, history of hypertension, total aortic arch replacement with elephant trunk implantation, preoperative D-dimer levels, SOFA score, serum creatinine levels, Glasgow Coma Scale, and blood urea nitrogen on the first postoperative day (**Supplementary Fig. 2**). The model was subsequently trained to predict outcomes based on these selected features.

### 3.3 TAAD Prediction Model

**Fig. 2.**
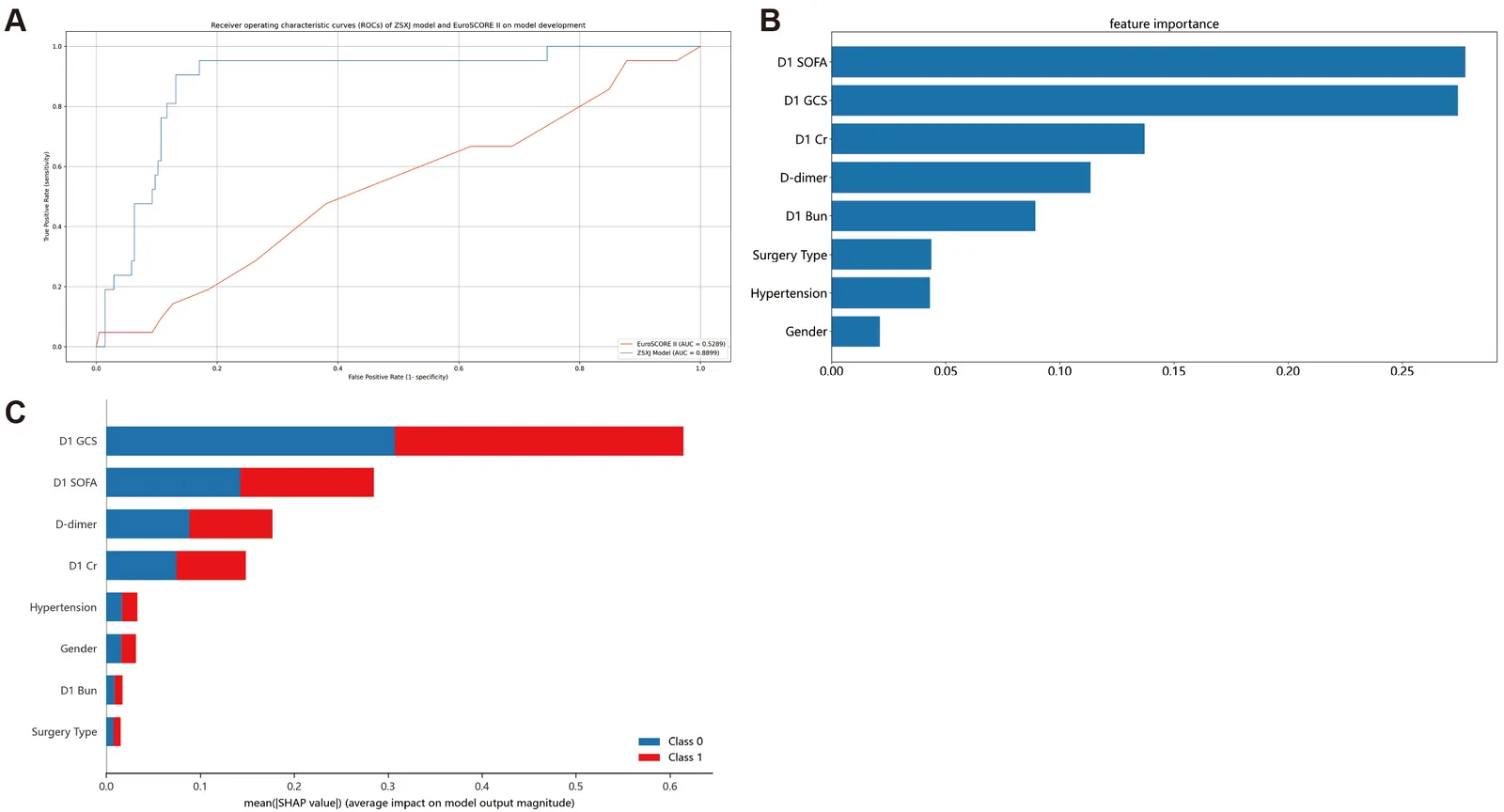
**Model development**. (A) Receiver operating characteristic (ROC) curves of the Zhongshan-Xijiao (ZSXJ) model and EuroSCORE II in the development cohort. (B) Feature importance of the ZSXJ model. (C) Feature contributions estimated using the SHapley Additive exPlanations (SHAP) values.

To assess the predictive performance of the random forest model, we performed a two-fold cross-validation (k = 2) on the training set. The data were split into two subsets, and the model was iteratively trained on one subset and validated on the other, alternating between training and validation roles. This procedure was used to assess the performance of the final model. The final model, which incorporated eight selected variables, achieved a notable AUROC of 0.8899 (95% CI, 0.7988–0.9469) (Table 2 and Fig. 2A). Sensitivity and specificity analyses were also performed. SHAP values were calculated for the internal testing set and are presented in Fig. 2B,C. Feature values are displayed on a spectrum, with blue indicating the lowest value. A positive SHAP value indicates an increased risk of in-hospital mortality. Features were ranked based on the sum of absolute SHAP values across all samples. Among all predictors, the SOFA score emerged as the most critical predictor of in-hospital mortality in the final model.

**Table 2.** **Characteristics of the developed model**.

	Deviation	External POD 1	External POD 2	External POD 3
AUROC	0.8899	0.8331	0.8341	0.8586
Best threshold	0.3313	0.0752	0.1631	0.0723
Gray zone	95%	95%	95%	95%
Values in the gray zone	(0.7988, 0.9469)	(0.7722, 0.8871)	(0.7597, 0.8979)	(0.7933, 0.9158)
F1 score	0.5263	0.3467	0.4204	0.3978
Precision	0.3636	0.2167	0.2946	0.2624
Recall	0.9524	0.8667	0.7333	0.8222
Sensitivity	0.9524	0.8667	0.7333	0.8222
Specificity	0.8293	0.6874	0.8248	0.7694
PPV	0.3636	0.2167	0.2946	0.2624
NPV	0.9942	0.981	0.9688	0.9775

POD, postoperative day; AUROC, area under the receiver operating characteristic curve; PPV, positive predictive value; NPV, negative predictive value.

### 3.4 External Validation and the Superiority of the Final Model

**Fig. 3.**
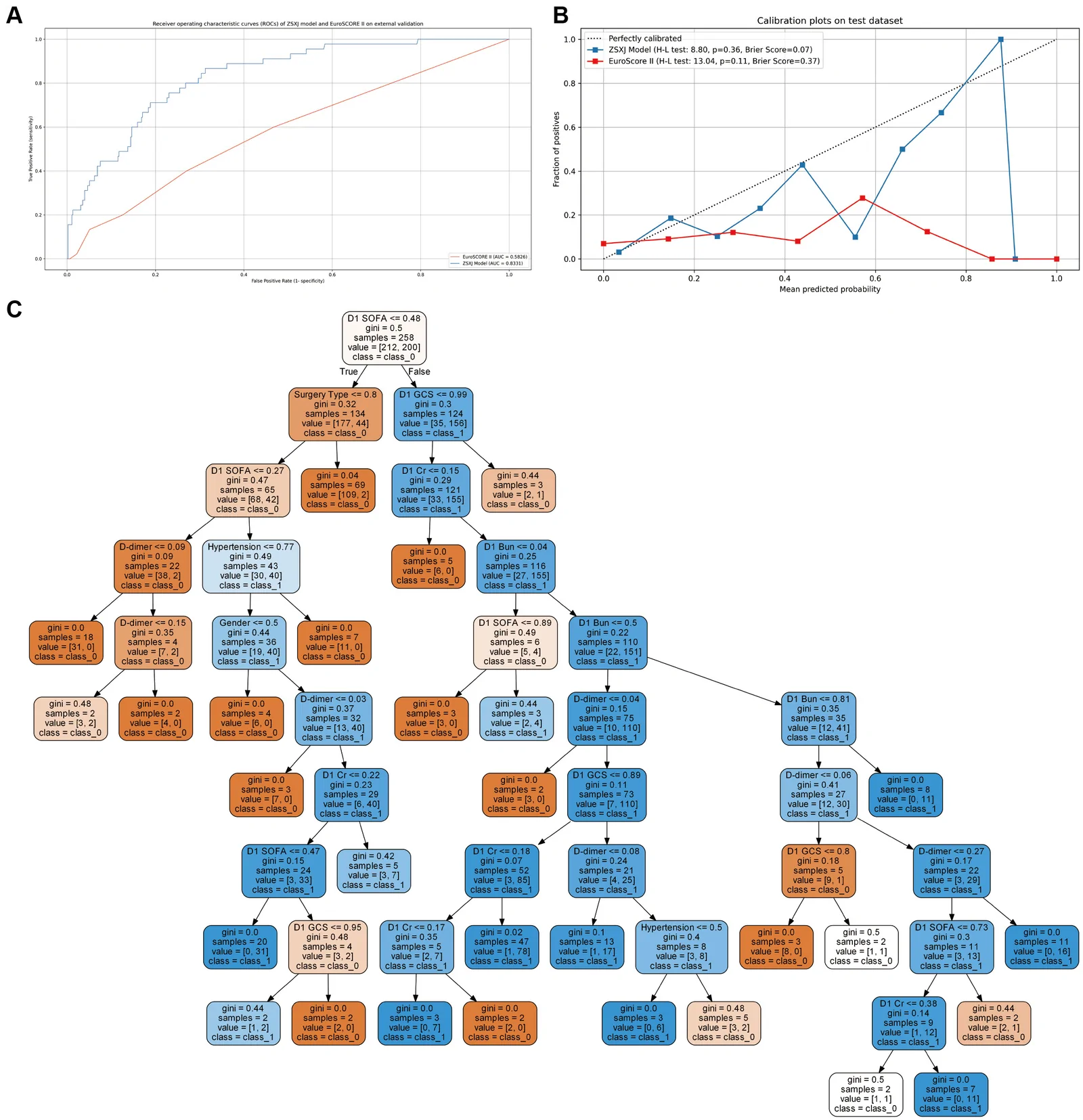
**External validation**. (A) Receiver operating characteristic (ROC) curves of the ZSXJ model and EuroSCORE II in the external validation cohort. (B) Calibration plots for the ZSXJ model and EuroSCORE II. (C) Decision tree of the ZSXJ model.

The results of the external validation are presented in Table 2 and Fig. 3. Our model exhibited strong generalization capabilities in the XJ cohort, with an AUROC of 0.8331 (95% CI, 0.7722–0.8871). Sensitivity and specificity analyses are also summarized in Table 2. To further explore the performance of our model, we generated a calibration plot (Fig. 3B) and a decision tree (Fig. 3C).

**Fig. 4.**
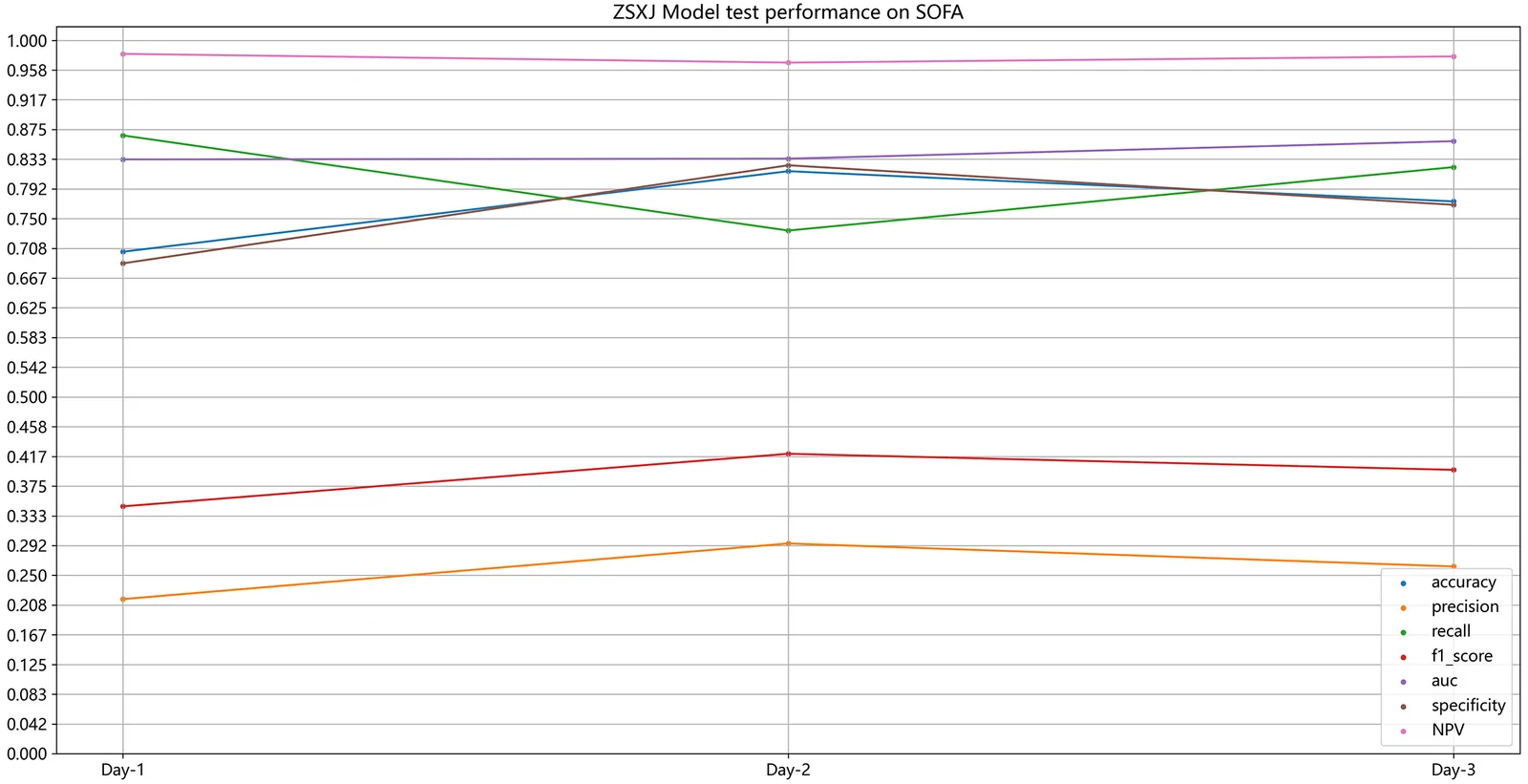
**Model validation using dynamic SOFA inputs**.

Given the dynamic nature of the SOFA score, which allows daily assessment, SOFA values from PODs 2 and 3 in the XJ cohort were utilized to replace the original POD 1 SOFA score employed during model development. With this modification, the model maintained an AUROC of 0.8341 (95% CI, 0.7597–0.8979) for POD 2 and 0.8586 (95% CI, 0.7933–0.9158) for POD 3 (Fig. 4).

Additionally, a comparison was conducted with the EuroSCORE II to evaluate the superiority of our model. The results indicated that our ZSXJ model achieved a significantly higher AUROC of 0.8899 (95% CI, 0.7988–0.9469) compared with the EuroSCORE II, which had an AUROC of 0.5289 (95% CI, 0.3916–0.6632; *p* = 0.001). This comparison highlights the enhanced predictive accuracy of our model.

## 4. Discussion

Despite numerous reports of predictive models for in-hospital mortality in TAAD, no consensus has been reached. Thus, this study developed and validated an accurate ML model using eight variables to predict in-hospital mortality for TAAD patients undergoing surgical repair. These variables reflect preoperative conditions and postoperative organ function, enabling accurate daily risk assessment during the early postoperative period. Indeed, by incorporating both preoperative and postoperative factors, this model demonstrated superior accuracy and specificity compared with existing prediction models.

Unlike other large registries, the proportion of patients undergoing total aortic arch replacement with elephant trunk was notably high in our cohorts, reflecting current preferences and standard clinical practice in mainland China. Furthermore, the postoperative SOFA score was identified as the most significant predictor in our model. The SOFA score, widely used in critical care to evaluate organ function [15], has been validated in prior research for its ability to predict mortality in TAAD patients, with SOFA values from the first three postoperative days strongly associated with in-hospital mortality [16,17]. Research indicates that aortic dissection and the ensuing surgical intervention can trigger a systemic inflammatory response syndrome, which is significantly associated with postoperative organ function impairment and mortality [18,19]. The SOFA score provides a quantitative measure of organ function, addressing weaknesses in other models. Additionally, postoperative acute kidney injury is a major complication following TAAD, while many studies have traditionally used preoperative serum creatinine levels as a predictive factor [20,21]. Our study found that creatinine levels on POD 1 were also critical independent predictors, enhancing the predictive accuracy of the model when integrated with other features. Consistent with previous reports, D-dimer and blood urea levels were also identified as factors associated with postoperative mortality [22]. This concordance with prior research underscores the utility of these biomarkers in assessing post-surgical outcomes.

Notably, EuroSCORE II and GERAADA scores do not permit continuous assessment of patients, which is critical because the condition of postoperative patients can fluctuate significantly. The GERAADA score, specifically designed for patients with TAAD, achieved an AUROC of 0.725, placing this metric at the lower end of the moderately accurate range [4]. Nežić et al. [5] found that, compared with the GERAADA score (AUC 0.55; 95% CI, 0.402–0.698), EuroSCORE II demonstrated superior discriminatory power for predicting mortality following acute TAAD surgery (AUC 0.799; 95% CI, 0.701–0.896). However, the GERAADA score lacks detailed laboratory data, specific descriptions of the onset and duration of malperfusion, and clear definitions of malperfusion for each organ [23]. The ZSXJ model developed in this study was externally validated using retrospective data from another center and demonstrated a high AUC, indicating strong predictive performance. Given the dynamic nature of postoperative conditions, a one-time scoring system may omit critical clinical information pertinent to assessing postoperative TAAD risk. Therefore, this study explored a dynamic model that uses SOFA scores from PODs 2 and 3 to provide a more accurate and timely evaluation of patient status.

### Limitations

This study has several limitations. While our model demonstrates high accuracy, the model relies on data extracted from electronic medical records, which are susceptible to missing data, errors, or omissions, especially for patients who died within 24 hours. Patients who received renal replacement therapy before surgery were not included. These discrepancies could introduce bias into the final predictions. Additionally, external validation was conducted at only one center, underscoring the need for broader prospective validation across multiple sites. Another limitation is the exclusion of preoperative imaging data and preoperative medications from the analysis. Incorporating these elements could provide a more nuanced understanding of patient conditions, although this approach is more complex to implement. Finally, a comparison with other models, such as the GERAADA score, was not feasible due to missing data, limiting our ability to benchmark the performance of our model against established tools. Further studies addressing these gaps are necessary to enhance the applicability and reliability of the model in clinical settings.

## 5. Conclusion

In conclusion, the present study identified eight key features and developed an ML model to predict in-hospital mortality among patients with TAAD undergoing surgical repair.

## Data Availability

The datasets generated and analysed during the current study are not publicly available due to institutional regulations but are available from the corresponding authors on reasonable request.

## References

[1] Pape LA, Awais M, Woznicki EM, Suzuki T, Trimarchi S, Evangelista A (2015). Presentation, Diagnosis, and Outcomes of Acute Aortic Dissection: 17-Year Trends From the International Registry of Acute Aortic Dissection. Journal of the American College of Cardiology.

[2] Conzelmann LO, Weigang E, Mehlhorn U, Abugameh A, Hoffmann I, Blettner M (2016). Mortality in patients with acute aortic dissection type A: analysis of pre- and intraoperative risk factors from the German Registry for Acute Aortic Dissection Type A (GERAADA). European Journal of Cardio-Thoracic Surgery.

[3] Nashef SAM, Roques F, Sharples LD, Nilsson J, Smith C, Goldstone AR (2012). EuroSCORE II. European Journal of Cardio-Thoracic Surgery.

[4] Czerny M, Siepe M, Beyersdorf F, Feisst M, Gabel M, Pilz M (2020). Prediction of mortality rate in acute type A dissection: the German Registry for Acute Type A Aortic Dissection score. European Journal of Cardio-Thoracic Surgery.

[5] Nežić DG, Živković IS, Miličić MD, Milačić PA, Košević DN, Boričić MI (2022). On-line risk prediction models for acute type A aortic dissection surgery: validation of the German Registry of Acute Aortic Dissection Type A score and the European System for Cardiac Operative Risk Evaluation II. European Journal of Cardio-Thoracic Surgery.

[6] Pollari F, Nardi P, Mikus E, Ferraro F, Gemelli M, Franzese I (2024). Comparison of 4 mortality scores for surgical repair of type A aortic dissection: a multicentre external validation. European Journal of Cardio-Thoracic Surgery.

[7] Ma M, Cao H, Li K, Pan J, Zhou Q, Tang X (2023). Evaluation of Two Online Risk Prediction Models for the Mortality Rate of Acute Type A Aortic Dissection Surgery: The German Registry of Acute Aortic Dissection Type A Score and the European System for Cardiac Operative Risk Evaluation II. Journal of Clinical Medicine.

[8] Angraal S, Mortazavi BJ, Gupta A, Khera R, Ahmad T, Desai NR (2020). Machine Learning Prediction of Mortality and Hospitalization in Heart Failure With Preserved Ejection Fraction. JACC. Heart Failure.

[9] Tseng PY, Chen YT, Wang CH, Chiu KM, Peng YS, Hsu SP (2020). Prediction of the development of acute kidney injury following cardiac surgery by machine learning. Critical Care.

[10] Vincent JL, Moreno R, Takala J, Willatts S, De Mendonça A, Bruining H (1996). The SOFA (Sepsis-related Organ Failure Assessment) score to describe organ dysfunction/failure. On behalf of the Working Group on Sepsis-Related Problems of the European Society of Intensive Care Medicine. Intensive Care Medicine.

[11] Chan HY, Liu M (2023). Interpretable Sub-phenotype Identification in Acute Kidney Injury. AMIA Annual Symposium Proceedings. AMIA Symposium.

[12] Spencer APC, Goodfellow M (2022). Using deep clustering to improve fMRI dynamic functional connectivity analysis. NeuroImage.

[13] Wu W, Ma X (2020). Joint learning dimension reduction and clustering of single-cell RNA-sequencing data. Bioinformatics.

[14] Soussi S, Sharma D, Jüni P, Lebovic G, Brochard L, Marshall JC (2022). Identifying clinical subtypes in sepsis-survivors with different one-year outcomes: a secondary latent class analysis of the FROG-ICU cohort. Critical Care.

[15] Seymour CW, Liu VX, Iwashyna TJ, Brunkhorst FM, Rea TD, Scherag A (2016). Assessment of Clinical Criteria for Sepsis: For the Third International Consensus Definitions for Sepsis and Septic Shock (Sepsis-3). JAMA.

[16] Luo MH, Luo JC, Zhang YJ, Xu X, Su Y, Li JK (2022). Early postoperative organ dysfunction is highly associated with the mortality risk of patients with type A aortic dissection. Interactive Cardiovascular and Thoracic Surgery.

[17] Huo Y, Zhang H, Li B, Zhang K, Li B, Guo SH (2021). Risk Factors for Postoperative Mortality in Patients with Acute Stanford Type A Aortic Dissection. International Journal of General Medicine.

[18] Margraf A, Ludwig N, Zarbock A, Rossaint J (2020). Systemic Inflammatory Response Syndrome After Surgery: Mechanisms and Protection. Anesthesia and Analgesia.

[19] Corral-Velez V, Lopez-Delgado JC, Betancur-Zambrano NL, Lopez-Suñe N, Rojas-Lora M, Torrado H (2015). The inflammatory response in cardiac surgery: an overview of the pathophysiology and clinical implications. Inflammation & Allergy Drug Targets.

[20] Eghbalzadeh K, Sabashnikov A, Weber C, Zeriouh M, Djordjevic I, Merkle J (2018). Impact of preoperative elevated serum creatinine on long-term outcome of patients undergoing aortic repair with Stanford A dissection: a retrospective matched pair analysis. Therapeutic Advances in Cardiovascular Disease.

[21] Bayram M, Duman ZM, Timur B, Yaşar E, Üstünışık ÇT, Kaplan MC (2023). Predictive value of age, creatinine, and ejection fraction (ACEF) scoring system for operative mortality in patients with Stanford type A aortic dissection. Indian Journal of Thoracic and Cardiovascular Surgery.

[22] Huang M, Lian Y, Zeng Z, Li J (2024). D-dimer, C-reactive protein and matrix metalloproteinase 9 for prediction of type A aortic dissection patient survival. ESC Heart Failure.

[23] Nezic D (2021). GERAADA score for the prediction of mortality rate in acute type A aortic dissection surgery. European Journal of Cardio-Thoracic Surgery.

