# Supplementary material for: A Machine Learning Prediction Model for In-Hospital Mortality in Postoperative Patients With Type A Aortic Dissection
Source: Rev Cardiovasc Med. 2026 Aug 27;27(8):49025. doi: 10.31083/RCM49025 (PMC13539989; doi:10.31083/RCM49025)
Supplement: Supplementary file 1 [file 2153-8174-27-8-49025-s1.zip › Supplementary Material.docx]

|  | Logistic Regression | Random Forest | XGBoost | MLP | KNN | SVM |
| --- | --- | --- | --- | --- | --- | --- |
| AUROC | 0.8690 | 0.8899 | 0.8783 | 0.8674 | 0.8028 | 0.8690 |
| Best Threshold | 0.6212 | 0.3313 | 0.3506 | 0.6248 | 0.4479 | 0.4582 |
| Gray Zone | 95% | 95% | 95% | 95% | 95% | 95% |
| Values in the gray zone | (0.7640, 0.9430) | (0.7988, 0.9469) | (0.7821, 0.9429) | (0.7621, 0.9420) | (0.7147, 0.8777) | (0.7607, 0.9436) |
| F1 score | 0.4800  (0.3100, 0.5977) | 0.5263  (0.3513, 0.6445) | 0.5862  (0.3783, 0.7097) | 0.4737  (0.3030, 0.5946) | 0.3953  (0.2528, 0.5273) | 0.4737  (0.3175, 0.6112) |
| Precision | 0.3333  (0.1961, 0.4546) | 0.3636  (0.2241, 0.4898) | 0.4595  (0.2727, 0.6250) | 0.3273  (0.1892, 0.4464) | 0.2615  (0.1515, 0.3823) | 0.3273  (0.2000, 0.4603) |
| Recall | 0.8571  (0.6295, 0.9566) | 0.9524  (0.7500, 1.000) | 0.8095  (0.5556, 0.9474) | 0.8571  (0.6295, 0.9565) | 0.8095  (0.6296, 0.9524) | 0.8571  (0.0.6922, 1.000) |
| Sensitivity | 0.8571  (0.6295, 0.9566) | 0.9524  (0.7500, 1.000) | 0.8095  (0.5556, 0.9474) | 0.8571  (0.6295, 0.9565) | 0.8095  (0.6296, 0.9524) | 0.8571  (0.0.6922, 1.000) |
| Specificity | 0.8244  (0.7739, 0.8775) | 0.8293  (0.7723, 0.8794) | 0.9024  (0.8585, 0.9447) | 0.8195  (0.7644, 0.8725) | 0.7659  (0.7073, 0.8238) | 0.8195  (0.7655, 0.8702) |
| PPV | 0.3333  (0.1961, 0.4546) | 0.3636  (0.2241, 0.4898) | 0.4595  (0.2727, 0.6250) | 0.3273  (0.1892, 0.4464) | 0.2615  (0.1515, 0.3823) | 0.3273  (0.2000, 0.4603) |
| NPV | 0.9767  (0.9506, 0.9944) | 0.9942  (0.9706, 1.000) | 0.9737  (0.9482, 0.9948) | 0.9767  (0.9503, 0.9944) | 0.9752  (0.9477, 0.9941) | 0.9824  (0.9583, 1.000) |

**Supplementary Table 1. Performance of different machine learning models.** AUROC=area under the receiver operating curve, MLP=multi-layer perceptron, KNN=k-nearest neighbors, SVM=Support Vector Machine, PPV=positive predictive value, NPV=negative predictive value.

|  | Overall N=983 | Survival  N=897 | Death N=86 | P-Value |
| --- | --- | --- | --- | --- |
| Age | 55.0 (46.0-64.0) | 55.0 (46.0-64.0) | 59.0 (49.2,66.0) | 0.036 |
| Male, n (%) | 739 (75.3) | 670 (74.9) | 69 (80.2) | 0.331 |
| BMI | 25.1 (23.0-27.7) | 25.0 (23.0-27.7) | 25.4 (23.4-28.3) | 0.344 |
| Hypertension, n (%) | 612 (62.4) | 558 (62.3) | 54 (62.8) | 1 |
| **Pre-operative values** |  |  |  |  |
| D-dimer | 8.6 (3.0-21.2) | 7.9 (2.8-20.3) | 18.8 (7.7-37.0) | <0.001 |
| cTnT | 0.014 (0.008-0.033) | 0.014 (0.008-0.032) | 0.020 (0.011-0.083) | 0.038 |
| Cr | 76.0 (61.0-104.0) | 75.0 (61.0-99.2) | 101.0 (70.0-142.0) | <0.001 |
| Hb | 131.0 (118.0-144.0) | 131.0 (119.0-144.0) | 129.0 (115.0-142.0) | 0.387 |
| TB | 16.6 (11.2-23.2) | 16.4 (11.1-22.8) | 18.8 (12.3-30.7) | 0.038 |
| **Surgical Information** |  |  |  |  |
| TAAR, n (%) | 905 (92.1) | 823 (91.8) | 82 (95.3) | 0.332 |
| CPB duration | 159.0 (140.0-186.0) | 157.0 (139.0-184.0) | 171.0 (149.2-207.0) | <0.001 |
| Aortic clamping duration | 87.0 (74.5-105.0) | 87.0 (74.0-105.0) | 89.0 (79.8-119.8) | 0.131 |
| DHCA duration | 18.0 (16.0-22.0) | 18.0 (16.0-23.0) | 17.0 (16.0-20.5) | 0.265 |
| **Values on POD 1** |  |  |  |  |
| EuroSCORE II | 8.2 (3.2) | 8.1 (3.1) | 8.8 (3.5) | 0.093 |
| GCS | 14.0 (2.1) | 14.2 (1.6) | 11.4 (3.9) | <0.001 |
| SOFA | 7.2 (2.8) | 6.9 (2.6) | 10.4 (2.9) | <0.001 |
| D-dimer | 14.6 (7.8-22.7) | 13.9 (7.6-22.0) | 22.0 (13.1-35.0) | <0.001 |
| BUN | 10.0 (7.7-12.7) | 9.9 (7.7-12.5) | 12.0 (9.3-16.5) | <0.001 |
| cTnT | 0.5 (0.3-0.8) | 0.5 (0.3-0.8) | 0.8 (0.5-1.7) | <0.001 |
| Cr | 110.0 (80.0-156.0) | 106.0 (79.0-148.0) | 166.0 (116.0-227.0) | <0.001 |
| Hb | 105.0 (95.0-114.0) | 105.0 (95.0-114.5) | 101.0 (90.5-113.0) | 0.012 |
| TB | 34.0 (22.7-48.4) | 33.9 (22.7-48.4) | 34.6 (24.4-51.55) | 0.453 |
| Lactate | 6.1 (3.1-10.0) | 5.9 (3.0-9.5) | 9.6 (5.2-14.3) | <0.001 |
| **Clinical Outcomes** |  |  |  |  |
| MV duration | 2.0 (1.0-3.3) | 2.0 (1.0-3.0) | 6.8 (4.0-10.8) | <0.001 |
| ICU duration | 5.2 (3.5-8.9) | 5.0 (3.3-8.0) | 8.0 (5.0-14.0) | <0.001 |
| Hospital duration | 15.7 (11.0-20.0) | 16.0 (12.0-21.0) | 8.8 (6.0-15.2) | <0.001 |

**Supplementary Table 2 Comparison between the survival group and the death group.** Cr=serum creatinine; Hb=hemoglobin; TB=total bilirubin; TAAR=total aortic arch replacement with elephant trunk implantation; CPB=cardiopulmonary bypass; ACD= aortic clamping duration; POD=postoperative day; EuroSCORE II=European System for Cardiac Operative Risk Evaluation II; SOFA= Sequential Organ Failure Assessment; GCS= Glasgow Coma Scale; BUN=blood urea nitrogen; MV=mechanical ventilation; ICU= intensive care unit

**Supplementary Figure 1** Flowchart of the study.

**Supplementary Figure 2** Heatmap showing correlation between features
